# Effect of Agave Fructan Bioconjugates on Metabolic Syndrome Parameters in a Murine Model

**DOI:** 10.3390/ph16030412

**Published:** 2023-03-08

**Authors:** Eduardo Padilla-Camberos, Javier Arrizon, Georgina Sandoval

**Affiliations:** 1 Medical and Pharmaceutical Biotechnology Unit, Center for Research and Assistance in Technology and Design of the State of Jalisco, A.C. (CIATEJ), Av. Normalistas No. 800 Col. Colinas de la Normal, Guadalajara C.P. 44270, Jalisco, Mexico; 2 Industrial Biotechnology Unit, Center for Research and Assistance in Technology and Design of the State of Jalisco, A.C. (CIATEJ), Camino Arenero 1227, El Bajío del Arenal, Zapopan C.P. 45019, Jalisco, Mexico; 3 LIBBA Laboratory, Industrial Biotechnology Unit, Center for Research and Assistance in Technology and Design of the State of Jalisco, A.C. (CIATEJ), Av. Normalistas No. 800 Col. Colinas de la Normal, Guadalajara C.P. 44270, Jalisco, Mexico

**Keywords:** hypertension, weight gain, oligosaccharides, prebiotics, lipase, acylation

## Abstract

Metabolic syndrome is a complex disorder that combines abdominal obesity, dyslipidemia, hypertension, and insulin resistance. Metabolic syndrome affects 25% of the world’s population. Agave fructans have shown positive effects on alterations related to metabolic syndrome, so some investigations have focused on their bioconjugation with fatty acids to increase their biological activity. The objective of this work was to evaluate the effect of agave fructan bioconjugates in a rat model with metabolic syndrome. Agave fructans enzymatically bioconjugated (acylated via food-grade lipase catalysis) with propionate or laurate were administered orally for 8 weeks in rats fed a hypercaloric diet. Animals without treatment were used as the control group, as well as animals fed with a standard diet. The data indicate that the group of animals treated with laurate bioconjugates showed a significant decrease in glucose levels, systolic pressure, weight gain, and visceral adipose tissue, as well as a positive effect of pancreatic lipase inhibition. These results allow us to demonstrate the potential of agave bioconjugates, particularly laurate bioconjugates, for the prevention of diseases associated with metabolic syndrome.

## 1. Introduction

Metabolic syndrome is a cluster of metabolic disorders associated with obesity and type 2 diabetes: insulin resistance, hypertension, hyperlipidemia, hyperglycemia, and cardiovascular disease [1,2]. The prevalence of metabolic syndrome fluctuates worldwide as there is a close association with age, sex, race/ethnicity, and the criteria used for diagnosis [3]. It is estimated that approximately 25% of the world’s adult population suffers from metabolic syndrome and that the probability of dying from its complications as well as suffering a stroke increases considerably year by year [4,5]. At present, the main origin of metabolic syndrome has not been established; however, genetic and epigenetic factors, as well as the accelerated lifestyle of individuals and the high caloric intake associated with visceral adiposity, are the main inducers in the development of the syndrome [6,7]. Treatment of people with metabolic syndrome consisting of implementing lifestyle and diet changes, and increasing physical activity can improve the individual components of metabolic syndrome, but reducing cardiovascular risk through treatment of atherogenic dyslipidemia should be addressed directly with medications [8].

To reduce the negative effects of metabolic syndrome on human health, the consumption of bioactive compounds and natural fibers from plants, such as antioxidants and prebiotics, has increased worldwide. In particular, for prebiotics, it is well known that the positive effects of fructans in metabolic syndrome and the biological properties of these polysaccharides depend on the fructosyl linkages inside them, thus they have been used in plant-derived products for the formulation of functional food products against metabolic syndrome.

These plant-derived products have played an important role in maintaining human well-being. For hundreds of years, since ancient times, natural products and their derivatives have been used, mainly in the development of pharmaceuticals for the treatment of human conditions [9]. A recent review by Wang et al. remarked on the potential of several polysaccharides obtained from plants for the treatment of metabolic syndrome, with mechanisms of action associated with the regulation of apoptosis, inflammation, and intestinal microbiota, among others. Such polysaccharides have mainly glycosidic bonds α-(1 → 6)-D, α-(1 → 4)-D, and β-(1 → 4)-D, and their biological activities are closely related to their primary and higher structures [10].

Other well studied groups of plant polysaccharides are inulin and fructans. Fructans from *Agave* genera differ from inulin in the type of linkage. Indeed inulin is lineal, while agave fructans are a complex mixture of fructooligosaccharides containing principally β (2 → 1) linkages, and some β (2 → 6) and branching moieties, leading to ramified structures [11,12].

Fructans from tequila agave (*Agave tequilana* Weber var. azul) have been shown to have positive effects on metabolic disorders associated with metabolic syndrome [13,14,15,16]. For example, studies evaluating the effects of fructans on glucose concentration in animal models have been consistently positive [17]. For instance, Castillo-Andrade et al. [18] evaluated the physiometabolic effects of *Agave salmiana* fructans as a dietary supplement in male Wistar rats. They found that the inclusion of 12.5% *Agave salmiana* fructans in the diet of the animals induced beneficial physiometabolic effects after the seventh day of treatment [18,19].

Functionalization of fructans, such as acylation with fatty acids has also been reported, for instance, for inulin by chemical esterification [20], and by enzymatic bioconjugation of *Agave tequilana* fructans [21]. Some esterified fructans have been tested on metabolic syndrome, but the results were inconclusive, showing an impact on food intake in some cases [20], and no impact on zoometric parameters in others [22].

Animal diet-induced metabolic models are commonly used to study metabolic syndrome because of their simplicity, accuracy, and low cost [23]. The animals most commonly used to develop metabolic syndrome are rats, mainly Wistar and Sprague-Dawley, because they manifest the characteristics of obesity, diabetes, and hypertension. For this study, such a model was chosen and metabolic syndrome was induced with specific diets formulated with fat and carbohydrates that involve a high caloric content [24]. Accordingly, in the present study, we established the metabolic syndrome model in young rats by including a hypercaloric diet and evaluated the effect of agave fructan bioconjugates orally administered on parameters associated with metabolic syndrome.

## 2. Results and Discussion

### 2.1. Synthesis of Agave Fructan Bioconjugates

**Figure 1:**
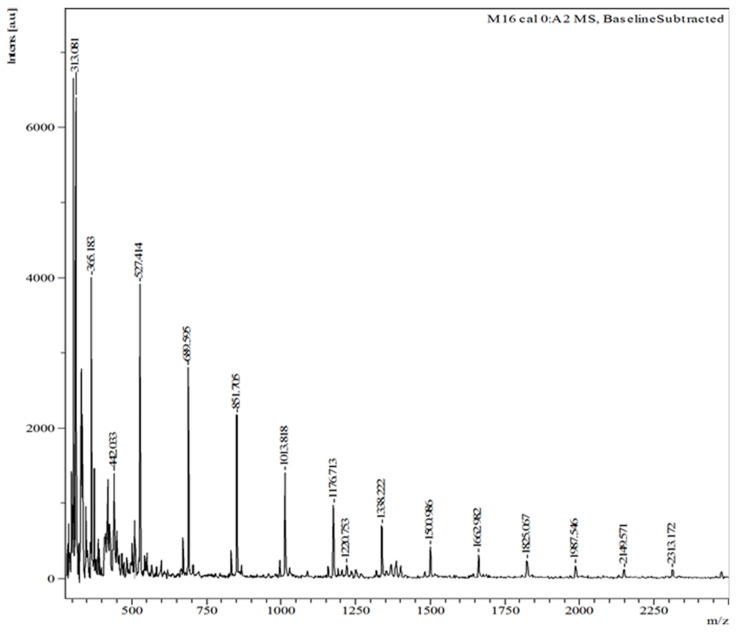
MALDI−TOF−MS profile of the FOS used in this study.

It was previously reported that when enzymatic acylation of agave fructans is performed, only short-chain fructans are acylated [21]; therefore, agave fructans enriched in fructooligosaccharides (FOS) were used. Figure 1 shows the Matrix-Assisted Laser Desorption/Ionization Time-Of-Flight (MALDI-TOF-MS) profile of the FOS used. The *m*/*z* distribution ranged from 527.37 to 1826.7, corresponding to FOS with a degree of polymerization (DP) from 3 to 11, respectively (Figure 1). FOS with a DP from 4 to 7 accounted for 53% mol of the total mixture and the most abundant FOS had a DP of 5 (14.8% mol). The mass distribution of this agave FOS mixture used for the acylation reactions is smaller than that of natural agave fructans with DPs ranging from 3 to 30 [10]. This reduction was caused by industrial processing, which favors agave FOS acylation. Indeed, it was previously shown that the immobilized lipase B from *Candida antarctica* Lipozyme^TM^ 435, preferably acylated FOS up to DP 8 [20]. This could be due to the fact that the access of large molecules to the catalytic site of the lipase is hindered due to steric effects [25].

Two kinds of agave fructan bioconjugates were prepared, with short-chain (propionate) and medium-chain (laurate) acyl groups. Both short- and medium-chain fatty acids have been described as beneficial for the colonic gut microbiota [26]. Indeed, the short-chain fatty acids (SCFAs) acetate, propionate, and butyrate are reported as important fuels for intestinal epithelial cells [6]. Regarding propionate, the results of Byrne et al. in nonobese men suggested that colonic propionate may play an important role in human appetitive and reward-based eating behavior [27]. Medium-chain fatty acids and triglycerides ingestion results in ketone body production, provoking a thermogenic response [28]. Lauric acid showed insulinotropic effects in mouse models [29]. Thus, these fatty acids were investigated as acyl donors for agave fructan bioconjugates.

**Figure 2:**
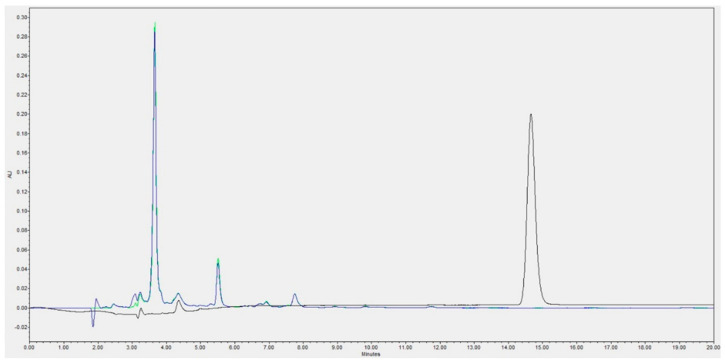
HPLC profile of the agave fructan bioconjugates with vinyl laurate at the beginning (black line) and at the end (green and blue lines) of the reaction.

The conversion of these two acyl groups to agave fructan bioconjugates was similar; as an example, Figure 2 shows the HPLC product profile of agave fructan bioconjugates synthesized with laurate. Fructans are not detected by the diode-array, and therefore, the visualized peaks are agave fructan bioconjugate products. Complete consumption of vinyl laurate (acyl donor) was observed. A similar behavior was detected with propionate agave fructan bioconjugates (data not shown).

**Figure 3:**
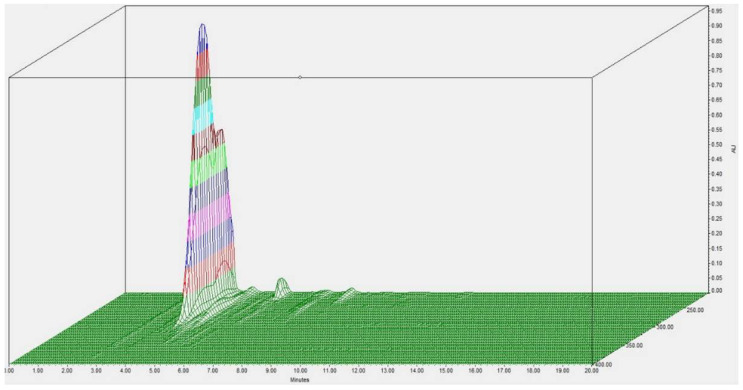
Three−dimensional chromatogram of agave fructan bioconjugates with laurate showing the variety of products due to agave fructan ramification.

Additionally, a complex range of agave fructan bioconjugate products is obtained (Figure 3), which is due to the acylation at different positions of the hydroxyl groups of the ramified agave fructans. In the same manner, when acylation was carried out with agave fructans with a higher DP, a complex mixture of agave fructan bioconjugates was also observed [20]. Therefore, the highly complex branched structure of agave fructans could cause acylation in hydroxyl groups at different positions [10,11], which could impart different bioactive properties when compared with linear fructans.

### 2.2. Effects of Acute Supplementation with Agave Fructan Bioconjugates on Metabolic Syndrome Parameters

#### 2.2.1. Induction of Metabolic Syndrome with a Hypercaloric Diet

Most of the parameters that were analyzed showed that the animals that received the hypercaloric diet (HD) developed characteristics related to the metabolic syndrome (Table 1 and Table 2). Feed and water consumption in the different experimental groups were as expected according to animal body measures (Table 1). As expected, feed consumption with standard diet (SD) was slightly higher, as HD is more satiating. The use of butter as the main source of fat in the preparation of the hypocaloric diet favored the induction of metabolic syndrome in the animals [24]. Although the scattering in the results is large due to the preparation of the hypercaloric diet in our laboratory, other authors, such as Leonardi et al. [30], also showed that there is considerable variability in animal models of metabolic syndrome. On average, animals treated with the propionate (HDFP) and laurate (HDFL) bioconjugates had lower feed ingestion than those treated with HD and hypercaloric plus fructans (HDF) diets. ST-Onge et al. also showed that medium-chain fatty acid triglyceride consumption by overweight men reduced their food intake [31].

#### 2.2.2. Metabolic Syndrome Prevention by Agave Fructan Bioconjugates

**Figure 4:**
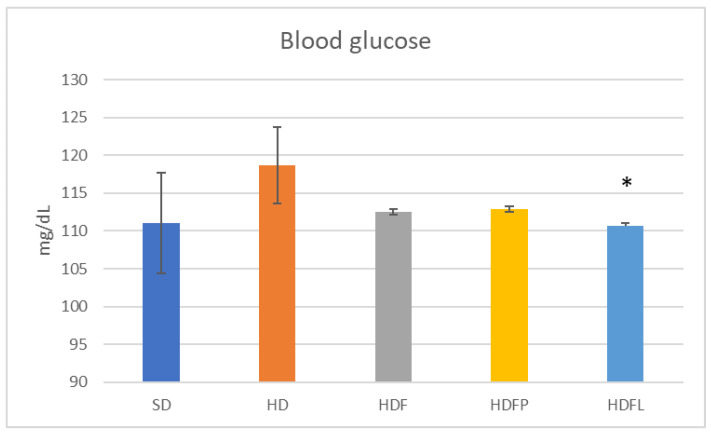
Blood glucose after eight weeks of treatment. Asterisks denote a significant difference vs. HD group (*p* < 0.05) after one-way ANOVA and Tukey tests. Error bars are ± standard deviation. Standard diet (SD), hypercaloric diet (HD), hypercaloric diet plus fructans (HDF), hypercaloric diet plus propionate-bioconjugated fructans (HDFP), and hypercaloric diet plus laurate-bioconjugated fructans (HDFL). * *p* < 0.05.

**Figure 5:**
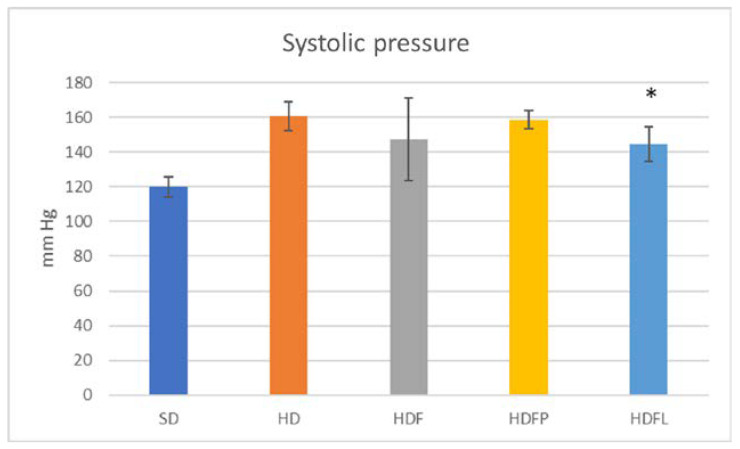
Systolic pressure after eight weeks of treatment. Asterisks denote a significant difference vs. HD group (*p* < 0.05) after one-way ANOVA and Tukey tests. Error bars are ± standard deviation. Treatment acronyms as in Figure 4. * *p* < 0.05.

**Figure 6:**
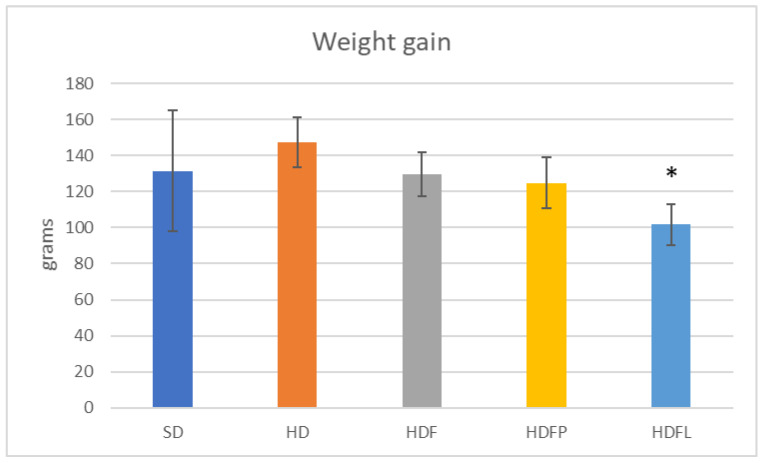
Weight gain after eight weeks of treatment. Asterisks denote a significant difference vs. HD group (*p* < 0.05) after one-way ANOVA and Tukey tests. Error bars are ± standard deviation. Treatment acronyms as in Figure 4. * *p* < 0.05.

**Figure 7:**
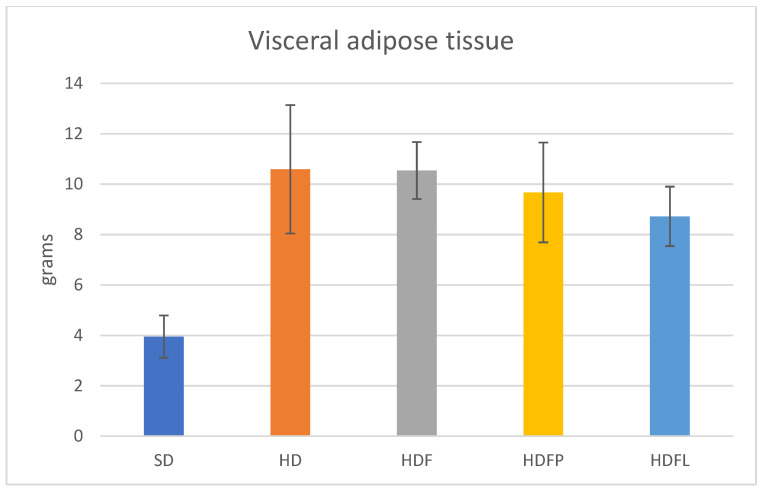
Visceral adipose tissue after eight weeks of treatment. Error bars are ± standard deviation. Treatment acronyms as in Figure 4.

**Figure 8:**
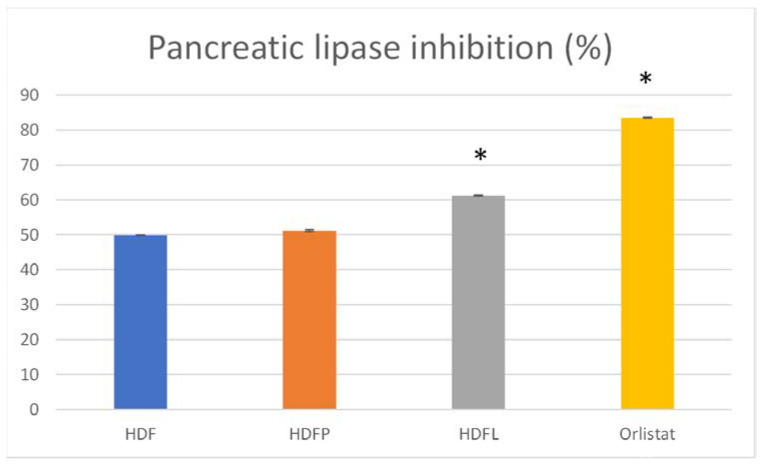
Pancreatic lipase inhibition. Orlistat (6 mg/mL) was used as a positive control. Fructans/agave fructan bioconjugates were evaluated at 1 mg/mL. Asterisks denote a significant difference vs. control (*p* < 0.05) after one-way ANOVA and Tukey tests. Error bars are ± standard deviation. Treatment acronyms as in Figure 4. * *p* < 0.05.

Table 2 and Figure 4, Figure 5, Figure 6, Figure 7 and Figure 8 show the biochemical and zoomorphic parameters after eight weeks of standard diet (SD), hypercaloric diet (HD), hypercaloric diet plus fructans (HDF), hypercaloric diet plus propionate-bioconjugated fructans (HDFP), and hypercaloric diet plus laurate-bioconjugated fructans (HDFL).

The animals that received the treatment with bioconjugates of the medium-chain acyl group (HDFL) showed a decrease in glucose levels when compared to the group with a hypercaloric diet (HD), (Figure 4). For the lipid profile parameters (Table 2), no significant differences were observed except for triglycerides where a significant decrease was observed in the HDFL group compared to the HD group; this effect was previously reported in a study with mice induced to obesity with a high-fat diet, which may be due to the proliferation of crypt cells related to lipid metabolism [19,32]. Previous studies have shown that agave fructans lower glucose levels in mice with obesity induced by a high-fat diet [13]. This reduction may be associated with the inhibition of enzymes related to carbohydrate metabolism, such as alpha amylase and alpha glucosidase, because preliminary studies of our working group demonstrated the postprandial hypoglycemic effect of agave fructans. Previous studies showed a significant 15% decrease in postprandial serum glucose values in C57Bl/6J mice fed a high-fat diet with agave fructans [16].

The HDFL sample group of animals showed a significant decrease in blood pressure (systolic) when compared to the HD group (Figure 5). Increased blood pressure is one of the parameters that is affected by high-fat dietary intake, which promotes the appearance of reactive oxygen species mediated by nuclear factor kappa B and proinflammatory cytokines, and activates the renin–angiotensin–aldosterone system, which is widely documented in the etiology of cardiovascular damage [33,34]. It has been reported that some types of soluble dietary fiber, including fructans, produce short-chain fatty acids by microbial fermentation and can regulate blood pressure [35,36]. Indeed, in a study conducted in rats with metabolic syndrome induced with a high fructose diet, it was found that supplementation with inulin-type fructans showed an antihypertensive effect [37].

Increases in body weight and body fat are among the main parameters related to metabolic syndrome. Different types of fructans have been studied to evaluate their potential as controllers of these parameters. Additionally, the animals that were treated with the HDFL bioconjugate exhibited significantly decreased weight gain, about 31% less than the HD group. The HDFP and HDF groups also showed a lower weight gain than the HD group; however, this difference was not significant (Figure 6). Visceral adipose tissue (Figure 7) was also about 18% lower in the HDFL group than in the group of animals fed an HD diet and the nonbioconjugated fructan group (HDF). These data show that the HDFL group significantly reduced weight gain and visceral adipose mass in HD-induced rats.

In healthy rats, no difference in body weight gain or adipose tissue weight was observed when fructans were supplemented in the diet [15]; however, in studies performed with mice of the C57Bl/6J strain fed fructans, a decrease in weight gain of the animals was demonstrated, and this effect was attributed to the induction of satietogenic peptides such as glucagon-like peptide-1 (GLP-1) [16]. It has been shown that rats treated with inulin-type fructans have increased serum levels of GLP-1, which, in addition to its satietogenic effect, inhibits macrophage inflammation [38]. Similarly, agave fructans were observed to prevent weight gain and hepatic steatosis in mice with high-fat-diet-induced obesity [13].

In clinical studies, agave fructans showed a beneficial effect on weight control, body fat and triglycerides in obese people during a 12-week treatment [14]. These effects may be due to the prebiotic activity of the fructans that promote the development of a healthy intestinal microbiota and therefore help to improve metabolic diseases [19,39]. Moreover, bioconjugates previously showed higher prebiotic activity than nonbioconjugated fructans by stimulating the growth of selected beneficial probiotic strains of the intestinal microbiota such as *S. boulardii*, *L. lactis*, *L. casei*, and *L. rhamnosus* [40].

Increases in body weight and body fat are among the main parameters related to metabolic syndrome. Different types of fructans have been studied to evaluate their potential as controllers of these parameters.

Unless pancreatic lipase degrades them, dietary fat is not directly absorbed from the intestine. Therefore, pancreatic lipase inhibitors have been studied as a treatment for obesity induced by a high-fat diet [41]. We selected orlistat, a well-studied pharmacological lipase inhibitor, as a positive control, which at 6 mg/mL gave almost 85% lipase inhibition (Figure 8), while the HDFL biconjugate at 1 mg/mL (used at this concentration because of its lower solubility) inhibited the lipase by 60%. This bioconjugate is a promising lipase inhibitor without the disadvantages of using orlistat, which has been associated with several mild-to-moderate gastrointestinal adverse effects [42].

Bioconjugation with propionate (HDFP) had a lesser effect on weight gain, visceral adipose tissue, and pancreatic lipase inhibition, similarly to Chambers et al. [20], who reported only weight and adiposity maintenance in acute supplementation with propionated inulin [20].

These results demonstrated that the bioconjugation of agave fructans has a positive effect on metabolic syndrome prevention, especially in the case of the medium-chain acyl group bioconjugate (HDFL).

## 3. Materials and Methods

### 3.1. Synthesis of Agave Fructan Bioconjugates

Agave fructan bioconjugates were enzymatically synthesized as described in Patent MX 358789 [40]. Commercially available organic agave fructans enriched in FOS, and Olifructine^TM^ (Nutriagaves, Guadalajara, Jal., Mexico) were used. The batch of Olifructine^TM^ had 53% mol of FOS of DP 4 to 7 (see Section 2.1 and Figure 1). The biocatalyst was the food-grade immobilized lipase B from *Candida antarctica* Lipozyme^TM^ 435 (Novozymes, Denmark, obtained through the broker Biotecsa, Mexico). Acylants (vinyl propionate and vinyl laurate), as well as solvents, were purchased from Sigma Aldrich (Burlington, MA, USA) at the highest purity available. Confirmation of acylation was performed by HPLC as reported previously [21], using a Luna^TM^ C18 column (Phenomenex, Monterrey, NL, Mexico) in a Waters Acquity^TM^ HPLC, using photodiode-array (PDA) detector at 217 nm; which makes it possible to detect the acylant substrates but not the fructans, with the advantage that the peaks observed once the acylant is consumed, correspond only to products. The mobile phase was methanol–water 90:10 at 0.6 mL/min. FOS are not completely soluble in the reactional system; however, once converted to bioconjugates, their solubility increased as the acylation reaction proceeded, as that kind of compound also has emulsification properties [21,25,43]. At the end of reaction, the immobilized lipase was filtered and the reaction solvent was eliminated in a Buchi^TM^ rotary evaporator. Purification of the bioconjugates was not needed, as the conversion of the limiting substrate (acylant) was almost 100% (see Figure 2), and unreacted fructans were insoluble in the reaction solvent. MALDI-TOF mass spectra analyses were performed in a Microflex LT, Bruker Daltonics system, using 2,5-dihydroxy benzoic acid (DHB) as the ionization matrix. Samples diluted (1:10 for propionate or 1:20 for laurate) were mixed with an equal part (*v*/*v*) of the matrix solution (10 mg/mL of DBH in ethanol–water; 1/1; *v*/*v*). Then, 1 mL of this mixture was deposited on the target plate and dried at room temperature. The equipment was calibrated from 380 to 3000 *m*/*z* with 1-kestose as the standard [43,44].

### 3.2. Diet-Induced Metabolic Syndrome Model

#### 3.2.1. Preparation of the Special Diet

The hypercaloric feed was formulated based on the AIN-93G diet and according to Cheng et al. [45]

The ingredients: cellulose, carboxymethyl cellulose, L-cystine, choline bitartrate, and butylhydroquinone were purchased from Sigma Aldrich (Burlington, MA, USA). Starch, clarified anhydrous butter, maltodextrin, sucrose, and soybean oil were purchased from a local feed formulating ingredient distributor. Vitamin and mineral premix were purchased from Dyets Inc. (Bethlehem, PA, USA) and casein was purchased from Hilmar Ingredients (Hilmar, CA, USA). The distribution in percentage is shown in Table 3, and macronutrient distribution compared to the standard diet is presented in Table 4. The hypercaloric diet was prepared by mixing all ingredients and baking at 100 °C for 1 h.

#### 3.2.2. Animals

Thirty male Wistar rats (3–4 weeks, 120 ± 20 g) were randomly housed in acrylic boxes, six animals per box, under standard environmental conditions (12 h artificial light/dark cycle) and water and food ad libitum. The number of animals was determined based on previous studies where 6 animals per group were used [46]. The animals were treated following the guidelines and requirements of the Declaration of Helsinki of the World Medical Association and the recommendations of the Mexican Official Standard for the Production, Care, and Use of Laboratory Animals (Secretariat of Agriculture, Livestock, Rural Development, Fisheries and Food (SAGARPA), Mexican Official Standard NOM-062-ZOO-1999). The protocol was favorably reviewed by the Internal Committee for the Care and Use of Laboratory Animals (CICUAL). Report Code: 2021-002A.

#### 3.2.3. Induction of Metabolic Syndrome and Treatment with Agave Fructans and Bioconjugates

Each box with animals was assigned a group: Group 1 was considered the control group, which continued to receive standard feed (SD) and did not receive treatment. Group 2 was replaced by a hypercaloric diet (own elaboration) and did not receive treatment (HD). Group 3 consisted of a hypercaloric diet and, in addition, a daily dose of 130 mg/kg of nonbioconjugated fructans (HDF) was administered. Groups 4 and 5 were treated with bioconjugates. Group 4 consisted of a hypercaloric diet and was given a daily dose of 130 mg/kg propionate-bioconjugated fructans (HDFP). The fifth experimental group was fed a hypercaloric diet and a daily dose of 130 mg/kg of laurate-bioconjugated fructans (HDFL) was administered.

Samples were administered daily for 8 weeks using an esopharyngeal cannula. The control group was administered phosphate-buffered saline (PBS). Samples were coded to maintain a blinded study.

The following parameters were measured during the study: zoometric data such as weight, height, and body mass index (not shown), as well as water and food consumption, were monitored daily. Food intake was calculated by subtracting the amount of residual food from the amount of supply food. Blood pressure was measured at week 7 by a noninvasive method using an occlusion cuff on the rat’s tail (Noninvasive blood pressure system, CODA-M1, Kent Scientific Corporation. Torrington, CT, USA). At the end of 8 weeks, all animals were fasted (12 h) and euthanized by decapitation according to the ethical guidelines described above. Blood was collected in a test tube and centrifuged at 3000× *g* for 15 min at 4 °C. At the same time, the visceral adipose tissue was excised, washed with 1X PBS solution (Sigma—Aldrich, Burlington, MA, USA), and weighed.

The serum obtained was stored at −80 °C for later use. Finally, glucose, cholesterol, triglycerides, LDL, and HDL parameters were measured in the stored serum using Randox kits (catalog numbers: GL2623, CH200, TR210, CH3841, and CH3811).

#### 3.2.4. In Vitro Study of Pancreatic Lipase Inhibition

Pancreatic lipase participates in the process of absorption of fatty acids from the diet and its inhibition reduces the levels of lipids such as cholesterol in the organism. The activity of porcine pancreatic lipase (type II) was measured by the addition of 1 mM 4-nitrophenyl butyrate as a colorimetric substrate to the fructan samples in 1 mM phosphate-buffered saline (PBS). They were incubated for 30 min at 30 °C and absorbance was measured at 405 nm. Orlistat (6 mg/mL) was used as a positive control based on the method of Kim et al. [41]. Fructans/agave fructan bioconjugates were evaluated at 1 mg/mL.

## 4. Conclusions

Our findings indicated that bioconjugation of agave fructans enriched in low DP FOS, particularly with medium-chain acyl groups, specifically laurate (HDFL bioconjugate), had a favorable effect on metabolic syndrome prevention. Although metabolic syndrome is a multifactorial condition, and its clinical approach requires integrated management with different pharmacological approaches, HDFL treatment has shown a positive impact on various parameters of metabolic syndrome, and therefore it is a promising dietary adjuvant to prevent metabolic syndrome. However, studies at the cellular and molecular levels are needed to elucidate the mechanisms of action of bioconjugates.

## 5. Patents

The processes described in our patent MX 358789 were used for the synthesis of agave fructan bioconjugates. A Mexican patent application for the results presented in this manuscript was presented with the number MX/a/2022/012414.

## Figures and Tables

**Table 1:** Feed and water consumption during the study.

Consumption ^1^/Diet ^2^	SD	HD	HDF	HDFP	HDFL
Water (mL/d)	23.48	16.74	14.89	13.59	12.66
Feed (g/d)	17.31	15.69	15.68	15.00	15.53

^1^ Milliliter per day (mL/d), grams per day (g/d). Values obtained from total water or feed consumed in each group divided by total days of study. ^2^ Standard diet (SD), hypercaloric diet (HD), hypercaloric diet plus fructans (HDF), hypercaloric diet plus propionate-bioconjugated fructans (HDFP), and hypercaloric diet plus laurate-bioconjugated fructans (HDFL).

**Table 2:** Biochemical parameters after eight weeks of treatment (mg/dL) ^1^.

Parameter ^2^/Diet ^3^	SD	HD	HDF	HDFP	HDFL
HDL	26.74 ± 5.02	28.43 ± 3.53	30.32 ± 5.64	27.69 ± 2.94	26.69 ± 2.45
LDL	22.71 ± 5.16	16.82 ± 7.37	9.02 ± 4.35	16.94 ± 6.00	16.07 ± 5.93
Total cholesterol	57.41 ± 10.17	53.95 ± 5.59	49.51 ± 5.81	56.86 ± 5.35	55.35 ± 5.07
Triglycerides	39.76 ± 13.25	43.45 ± 13.9	50.84 ± 16.07	61.19 ± 19.91	62.96 ± 19.91 *

^1^ Asterisks indicate statistically significant differences vs. HD group (*p* < 0.05) after one-way ANOVA and Tukey tests. Values are mean ± standard deviation. ^2^ HDL, high-density lipoprotein; LDL, low-density lipoprotein. ^3^ Standard diet (SD), hypercaloric diet (HD), hypercaloric diet plus fructans (HDF), hypercaloric diet plus propionate-bioconjugated fructans (HDFP), and hypercaloric diet plus laurate-bioconjugated fructans (HDFL). * *p* < 0.05.

**Table 3:** Distribution of components of the hypercaloric diet.

Component	Percentage
Corn starch	2.448
Anhydrous butter (clarified)	32.00
Casein	23.50
Maltodextrin	16.50
Sucrose	9.00
Mineral premix AIN-93G	4.59
Soybean oil	3.5
Microcrystalline cellulose	3.25
Carboxymethyl cellulose	3.25
AIN-93 vitamin premix	1.31
L-cystine	0.39
Choline bitartrate	0.26
Butylhydroquinone	0.002

**Table 4:** Macronutrient distribution in standard (SD) and hypercaloric (HD) diets.

Macronutrient	SD ^1^	HD
Fat	13.6	58.9
Carbohydrates	60.0	26.25
Proteins	26.0	14.85

^1^ The standard diet was acquired from Scientific diets (France), catalog number SAFE A30.

## Data Availability

Data is contained within the article.
